# The Ageing Body, Memory-Loss and Suicide in Georgian England

**DOI:** 10.1093/shm/hkab048

**Published:** 2021-08-01

**Authors:** Ella Sbaraini

**Keywords:** ageing, body, suicide, memory-loss, Georgian

## Abstract

This article examines why older people were particularly prone to suicide in Georgian England, and argues that their suicidality is best understood through the lens of the ‘ageing body’. By centring on the experiences of the suicidal, it proposes that suicide was not ‘medicalised’ in the way traditionally described in the historiography, being reconceptualised, over the course of the eighteenth century, as a product of lunacy. Instead, it contends that older people often thought that their suicides were a rational response to the struggles of ageing, which included physical decline and embodied memory-loss. To do this, it uses previously unseen coroners’ inquests from across England, in addition to wills and medical writings. It examines these inquests for what they can tell us about the emotional and embodied experiences of older suicidal people, thus contributing to an under-researched aspect of the social history of ageing.

On one August morning in 1795, Edward Hearne hanged himself in his bedroom. As his wife explained at his inquest, he had been ‘melancholy and Dull’ for some time, mostly on account of his age and infirmity—he was, indeed, aged 74, with disturbed eye-sight and a wavering memory. Due to his age and frailty, Hearne began to withdraw from his formerly independent, active life as an upholsterer, and he ‘often Complained & sayed being an old man he was afraid him & [his wife] would come to want as he was not so able as he used to be to work & get his living’.^1^ He was also, in his growing mental infirmity, unwittingly withdrawing from normal social life and communication; whenever his wife asked him a question, there was ‘a Considerable time before he gave any answer & sometimes when he did it was foreign to the question’.^2^ Hearne was acutely aware of this deterioration in his physical and mental health. He even asked his wife to help ‘get him into the Workhouse or to Bedlam or any Madhouse whatever for a Month to get his Head to right’.^3^ His inquest highlights experiences of loss, infirmity and mental and physical decline, all of which were extremely common among older suicidal people in eighteenth-century England. These individuals, and their complex and troubling stories, have been overlooked in a historiography which has not only neglected the relationship between suicide and age, but the embodied and emotional experiences of those who killed themselves.

Although Victor Bailey has used the lifecycle as a key framework for understanding Victorian ‘self-murder’, no historian has built on his insights to give age the attention and analytical importance it deserves in their studies of pre-Victorian suicide.^4^ In their seminal *Sleepless Souls*, Michael Macdonald and Terence Murphy devoted no more than nine pages to discussing age demographics, while R. A. Houston gave age only the most fleeting notice in *Punishing the Dead*.^5^ This is despite the fact that there were very significant differences in suicide prevalence among different age groups, with Macdonald and Murphy themselves acknowledging that older people were ‘unusually suicidal’ in early modern England.^6^ In Norwich, of 304 age-identified suicides from 1710 to 1815, over 40 per cent were aged 50 or above, and nearly 30 per cent were aged sixty or over.^7^ In a much smaller sample of Chelmsford inquests, over 40 per cent were also aged 50 and above.^8^ This preponderance towards suicide is even more pronounced if one considers the youthful demographic structure of eighteenth-century England; less than 20 per cent of people were aged over 50.^9^

As indicated, I will here be defining ‘older’ people as those aged 50 and above. Certainly, there was no ‘universal threshold of old age’, and people could pass into the final stages of the lifecycle at many different points, depending on a range of corporeal and social factors.^10^ However, people understood that those aged 50 and over were entering the latter stages of life, and often occupied visibly ageing bodies.^11^ As the popular *A guide to health* (1744) stated, ‘the fiftieth year’ is ‘where Old Age begins’.^12^ Thomas Paine, in his discussion of poor-law pensions, similarly saw 50 as ‘the approach of old age’, and as a time when ‘bodily powers … are on the decline’.^13^ Crucially for our purposes, in coroners’ inquests too, people in their fifties were regularly identified as ‘old’ or ‘elderly’ by neighbours and acquaintances. Mary Fenton, who died in 1791, was ‘an Elderly Woman, [who] appeared to be above fifty years of Age’.^14^ Mary Pritchard, who died in 1798, was also ‘an elderly Woman … [her neighbour] heard her say she was about fifty one years of age’.^15^ Undoubtedly, there are finer gradations within agedness, particularly between a ‘green old age’ and a ‘decrepit old age’.^16^ As we will see, some of the most painful examples of memory-loss and sensory decline occurred among those in later, ‘decrepit’ old age. These finer distinctions will be highlighted where possible but, given the lack of systematic recording of age-data in these sources (discussed below), this article looks at the over-50s as a whole. For 50 is a reasonable threshold to allow us to include those in the late autumn of life, many of whom appeared to be old, and struggled with the difficulties of ageing.

These age-related difficulties were very often linked to older people’s suicidality.^17^ It is a key contention of this article that the experiences of older suicidal people cannot be understood without a genuine appreciation of the physical, mental and social struggles of inhabiting an ageing body. The only way to achieve this is by paying attention to those who actually experienced suicidality, something rarely done in the historiography of this period.^18^ Macdonald and Murphy’s seminal thesis was, centrally, a metanarrative about a shift in legal and cultural positions on suicide. Their findings—that *non compos mentis* verdicts increased over the early modern period, and their argument, that this was due to the medicalisation and secularisation of suicide—were all products of their keen focus on ‘changes in attitudes and responses’ to suicide.^19^ Most subsequent Anglophone histories have not only responded to their work, but assumed its agenda. Houston’s most recent book was ‘the result of two decades of mulling over’ *Sleepless Souls*, similarly concentrated not on the ‘meaning of suicide’ to ‘the people who die’, but on ‘the attitudes and behaviour of those who interact with the self‐murderer after death’.^20^ By exploring, instead, the perspectives of the suicidal, we shift not only the focus, but the narratives told about eighteenth-century suicide. For, while Macdonald and Murphy used the long-term change in official coronial verdicts to conclude that, by the end of the century, suicide had been ‘medicalised’—that is, reconceptualised as ‘an insane action’ by physicians, legal practitioners and jurors—this article will use the details divulged in the coronial depositions to show that older people often viewed their suicides as a rational response to the struggles of ageing.^21^ Of course, Macdonald and Murphy are not wrong in arguing that juries ‘slowly adopted the medical explanation for acts of self-destruction’ and ‘excused suicides as innocent lunatics’; in the inquest sample used here, 97.2 per cent of people were formally given a verdict of insanity, or *non compos mentis*. However, this is not how many suicidal people viewed themselves; for them, suicide could be a logical and rational choice. Macdonald and Murphy have, therefore, only told us one side of the story.^22^

By focusing on the struggles of older suicidal individuals, this article contributes not only to histories of suicide, but histories of age and ageing. For although scholars such as Susannah Ottaway, Pat Thane and Lynn Botelho have thoughtfully examined many experiences of ageing and agedness, ‘old-age mental disorders and old-age emotional worlds still constitute an unexplored field’.^23^ It also, crucially, proposes that coroners’ inquests present rich scope for examining old-age struggles with pain, illness and memory-loss, and exploring how older people sought medical advice for these issues before turning to suicide. Even if suicide was not, as an act, always ‘medicalised’ in the eyes of the elderly suicidal, they often called upon medical practitioners to help with pre-suicide problems and illnesses.

This article is based upon a sample of 106 inquests (with depositions) into the suicides of older people, found in archives in Maidstone, Whitehaven, Chelmsford, Ipswich, Norwich, Bath, Middlesex, Westminster and the City of London.^24^ This sample derives from a larger database of 716 inquests located in these archives. They all, most likely, concern older people. As most inquests did not systematically record the ages of suicides, I have been extremely conservative in identifying individuals who were 50 or over, given indications in the material.^25^ Inquests were taken in England to investigate ‘suspicious’ deaths, and were conducted very soon after a fatality.^26^ They were ‘a local and participative event’ held either in the place where the body was discovered, or in a nearby alehouse or workhouse.^27^ They were overseen by a coroner—usually one of the local gentry or a legal professional—who was elected by local freeholders but often appointed to the office for life.^28^ The coroner summoned a jury to examine the body and hear witnesses.^29^ These witnesses, who were usually the deceased’s family, friends, neighbours, apothecaries or physicians—being, frequently, those who were present at the scene or aftermath of the death—were given a warrant by the coroner to give evidence, and their depositions were documented.^30^ Though it was not compulsory to seek medical testimony, medical practitioners quite often gave evidence, but mainly in their capacity as a person who had been advising or administering care to the deceased before they killed themselves.^31^ Their testimony was certainly given weight—if called, medical practitioners usually gave evidence first, and it was often lengthy—but the lack of ‘any legislative provision for the payment of medical witnesses’ until 1836 meant that autopsies, or extensive medical discussions about the cause of death, were uncommon.^32^

As Tim Hitchcock, Sharon Howard and Robert Shoemaker contend, depositions were recorded in ‘a form that was near to the actual words spoken’ by witnesses.^33^ Undoubtedly, they were not *exact* textual replicas of the verbal back-and-forths between the coroner, witnesses and jurors. The coroner’s and jurors’ questions were never recorded—though it is often clear what they were. Although they were guided by the questions, witnesses had the freedom to disclose a wide variety of details about the deceased, and often sought to report, verbatim, words or phrases the deceased had used, often documented in speech marks. Overall, depositions offer us fruitful evidence of how people acted and felt before their deaths, most especially in suicide cases, where legal significance was placed on someone’s mental state. Partly for this reason, this evidence presents some problems. Since suicide was illegal, depositions were not only used to ascertain why someone died, but whether the person was a *felo de se—*a rational individual liable for punishment—or *non compos mentis*. Therefore, most deposers, often having been close to the deceased, stressed their mental affliction. However, by and after the 1770s—the time from which most of our inquests come—well over ninety per cent of suicides were decreed *non compos mentis*, even on tentative accounts of mild distress.^34^ Thus, the need to *persuade* a coroner of mental affliction was minimal.

Furthermore, the legalistic necessity to highlight distress does not negate its existence. Within one inquest, there are often many detailed and cross-corroborated reports of a person’s behaviour and words. These second-hand accounts offer us unrivalled insight into older—and mostly poor and illiterate—suicidal people’s struggles and emotions. Within this sample, of the ninety-two people with identifiable occupational or social status, 61 per cent were lower-class, 28 per cent were of middling status, and 11 per cent were upper-class.^35^ Inquests are ‘one of the great neglected sources of English social history’ and, although some imaginative work has recently employed them, they have been underused by historians of emotion and mental health.^36^ That said, it should be noted that their potential as documents for exploring the experiences of the suicidal has been well established. These ‘valuable records’ were the basis for Olive Anderson’s chapter on ‘Experiences’ in her seminal work on Victorian and Edwardian suicide.^37^ An unbroken run of inquests in Victorian Hull were also used by Victor Bailey to look at ‘the experiences of those who committed suicide’ in the city.^38^ Similarly, John Weaver has used them to look at the psychological and emotional experiences of the suicidal in twentieth-century New Zealand.^39^ As Vera Lind, working on early modern Germany, notes, the ‘second-hand views’ captured in such court documents ‘are often the only clues of how a potential suicide might have felt before the act’, and ‘can function as a mouthpiece for those who could no longer speak and left no written statement about their acts’.^40^

Undoubtedly, these experiences are refracted through the voices of others, and their views—about suicide, about ‘sanity’, about relationships—are also captured in the sources, and are present in this article. However, just as, in the present day, ‘psychological autopsies’ (in which interviews with family members and health professionals are used to determine the background and potential reasons for someone’s suicide) are valued for offering ‘retrospective information’ on the ‘process of suicide’ for an individual, eighteenth-century coroners’ inquests can help us explore suicidal experiences in another era, particularly when explored collectively, and complimented with other lines of evidence (such as medical texts and wills).^41^

In exploring them, this article also builds upon the wider historiography of emotions which, as Rob Boddice notes, is embedded within research into ‘experience’.^42^ In particular, there are points at which we see older suicidal people struggling to remain rooted within what Barbara Rosenwein famously termed their ‘emotional communities’, the ‘group[s] in which people have a common stake, interests, values and goals’, which are constituted by ‘constellations’ of normative emotions.^43^ For, especially when dealing with memory-loss and a withdrawing social presence, older people could exhibit emotional behaviours which were alienating to those around them.

This article will first explore emotional responses to experiences of physical decline and deterioration. Secondly, it will examine issues around embodied memory-loss. Thirdly, it will consider feelings of burdensomeness, particularly anxieties about being a burden, and ideas about the burdensomeness of life itself. Lastly, it will look at the notion of being a withdrawn or withdrawing presence, and discuss aloneness and communication difficulties. Of course, these themes were often overlapping and interrelated, as shown in Table 1, which depicts all of the individuals whose inquests are quoted and discussed in the course of this article.^44^

**Table 1.** Inquests quoted in this article. Source: Inquisitions in Bath Record Office, 1782–1815, BC/4/1/1; Essex Record Office, 1797–1802, D/B 2/OFF3; Suffolk Record Office, 1792–1815, HB/10/9; London Metropolitan Archives, 1788–1799, LMCLIC65001-12; Westminster Muniment Room, 1762–1799, WACWIC65202-39; Kent History and Library Centre, 1769–1816, Md/JCi1769-1816; Norfolk Record Office, 1710–1815, NCR 6a; Whitehaven Archive and Local Studies Centre, 1718–1803, D/LEC/CRI

Year of death	Name	Gender	Issues described in the inquest	Source reference
1795	Edward Hearne	Male	Physical decline, occupational/economic problems, memory-loss	LMA, LMCLIC650080410
1802	Thomas Browne	Male	Physical decline, occupational/economic problems	ERO, D/B 2/OFF3/50
1797	Isaac Hendley	Male	Physical decline, occupational/economic problems, memory-loss	LMA, LMCLIC650100204
1790	William Richmond	Male	Physical decline, occupational/economic problems	WAMR, WACWIC652300490-1
1792	James Nicholas	Male	Physical decline, occupational/economic problems	SRO, HB/10/9/7/3
1774	Cuthbert Cousins	Male	Physical decline, occupational/economic problems	WAMR, WACWIC652120185
1799	Thomas Emperor	Male	Physical decline, occupational/economic problems	LMA, LMCLIC650120531
1771	Thomas Norman	Male	Physical decline	WAMR, WACWIC652110247
1803	John Braithwaite	Male	Physical decline, memory-loss, anxiousness	WA, D/LEC/CRI/112/13
1815	Jesse Cook	Male	Physical decline, memory-loss	SRO, HB/10/9/30a/12
1783	George Heming	Male	Physical decline, memory-loss	WAMR, WACWIC652230531-2
1776	James Barkley	Male	Memory-loss	WAMR, WACWIC652160520
1772	Robert Lovell	Male	Memory-loss, occupational/economic problems	WAMR, WACWIC652120295-6
1792	Martha Fuller	Female	Memory issues	WAMR, WACWIC652320753
1803	James Dowdle	Male	Burdensomeness	BRO, BC/4/1/2/91-93
1768	James Hilton	Male	Burdensomeness	WAMR, WACWIC652080122
1777	Sarah Fenwick	Female	Burdensomeness, occupational/economic problems	WAMR, WACWIC652170357
1804	Thomas Coe	Male	Burdensomeness, occupational/economic problems	ERO, D/B 2/OFF3/54
1779	Phillis Jury	Female	Burdensomeness	KH&LC, Md/JCi/80
1763	Ann Tiffin	Female	Burdensomeness	WA, D/LEC/CRI/72/4
1718	Robert Lowthian	Male	Burdensomeness	WA, D/Lee/CR I, 27/4
1804	Margaret Hay	Female	Physical decline, withdrawing presence, ‘childishness’	WAMR, MS: no. 55
1788	William Woodcock	Male	Physical decline, withdrawing presence, ‘childishness’	LMA, LMCLIC650010230-1
1813	Elizabeth Sharp	Female	Withdrawing presence	WAMR, MS: no. 23
1799	John Bucknell	Male	Withdrawing presence	LMA, LMCLIC650120605
1799	Robert Rose	Male	Physical decline, withdrawing presence	SRO, HB/10/9/15/14
1804	William Buttram	Male	Withdrawing presence, anxiousness	SRO, HB/10/9/20/18
1772	Elizabeth Hughes	Female	Physical decline, withdrawing presence, anxiousness	WAMR, WACWIC652120246
1783	Margaret Leverett	Female	Withdrawing presence, anxiousness	ERO, D/B 2/OFF/3/29
1776	Joseph Fisher	Male	Physical decline, anxiousness	WA, D/LEC/CRI/85/1
1792	John Abbott	Male	Anxiousness, delusion	LMA, LMCLIC650050375

Overall, this article will argue that older people’s preponderance towards suicide in this period is best understood through the lens of the ageing body. It will show that, by repositioning our attention on the lives of the suicidal, rather than upon the views of those who outlived them, a more complex history of ideas about mental health emerges. While some older people felt mentally unwell before their suicides, others saw their self-inflicted deaths as a rational response to the struggles of pain, deterioration and dependency in old age.

## Physical Decline

**Fig. 1.**
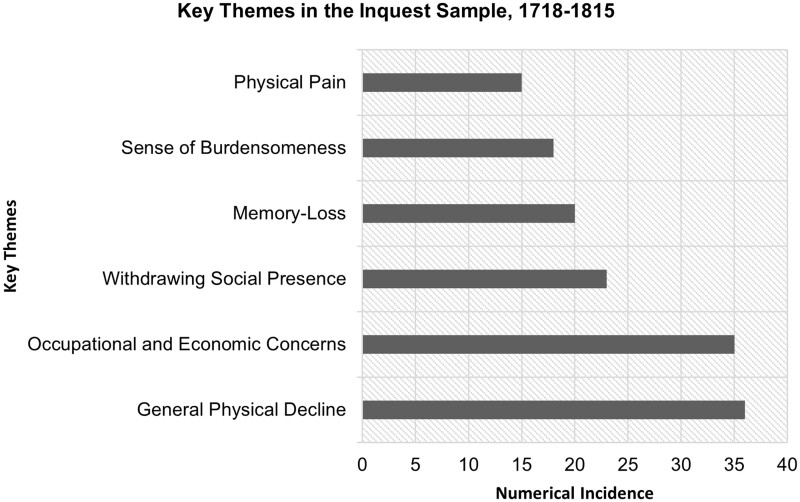
Incidence of key themes in the inquest sample, 1718–1815. Source: Inquisitions in Bath Record Office, 1782–1815, BC/4/1/1; Essex Record Office, 1797–1802, D/B 2/OFF3; Suffolk Record Office, 1792–1815, HB/10/9; London Metropolitan Archives, 1788–1799, LMCLIC65001-12; London Metropolitan Archives, 1747–1786, LMCOIC65102; Westminster Muniment Room, 1762–1799, WACWIC65202-39; Kent History and Library Centre, 1769–1816, Md/JCi1769-1816; Whitehaven Archive and Local Studies Centre, 1718–1803, D/LEC/CRI

In Thomas Knagg’s *A sermon against self-murder* (1708), Knagg declared that it was always wrong to kill oneself, even if our ‘many Years’ are rendered ‘irksome and tedious by reason of the many miseries Old Age exposeth us to’, such as ‘the Stone’ and ‘the Gout’.^45^ As the ‘Rationalist’ similarly contended in 1774, people should never ‘quit the world’ and the ‘prison of the body’, even if it was ‘broken with old age’.^46^ There were, then, concerns about the suicidality of those facing deteriorating physical health, and with good reason. Kevin Siena has noted the significance of somatic struggles among the suicidal in this period, arguing that suicide was an ‘illness strategy’ for desperate people.^47^ Siena has not, however, looked at the issue of age, and at the fact that bodily pain and infirmity were especially common among older suicidal individuals.^48^ As shown in Figure 1, within the inquest sample, concerns about physical deterioration were significant, as were anxieties about occupational or economic vulnerability, which—as discussed below—were often deeply interrelated. It should be noted that older people often talked not merely of their physical pain or distinct bodily problems, but in general terms of deteriorating health. Thus, we need to think in terms of processes of decline, and of the temporally situated experience of looking back at a time of better bodily health, and forward to a time of ever-worsening wellbeing.^49^

These worries were clearly articulated by Thomas Browne, a 60-year-old man who hanged himself in Saffron Walden in 1802. The day before he died, he complained to a neighbour that ‘he was not the man he had been, meaning … that age and Infirmity had reduced him’; he even believed—to the confusion of his neighbours—‘that he had got no Legs’, a delusion which potentially expressed anxieties about his ailing body. As one neighbour explained, he saw Browne:

look wistfully at him, [so he] asked him whether he had got the ague … he said, no, he had lost his legs, he had got no Legs, & this deponent says that he shortly afterwards said to the best of his Recollection, that he should not want them for he was going soon either to Heaven or Hell but Hell he expected.^50^

Browne’s legs seemed to ground him in the living world, with Browne determining that his ‘lost’ legs would not be needed in the afterlife. He apparently located a sense of death’s inevitability in his declining capacity to walk and work as a labourer. A neighbour explained that, when he came to her house equipped with a shovel, she had asked him where he was going, to which ‘he said “ you will never see me any more” & she asked him whether he was going on the Tramp [i.e. walking about searching for work] & he said he would never go upon the Tramp any more’.^51^ In declaring that he ‘would never go upon the Tramp any more’, Browne was apparently making a statement about his future work-plans but, when coupled with his contention that ‘you will never see me any more’, these work-plans, and his mortality, were seemingly conflated. Undoubtedly, some ambiguity exists in the statements reported by Browne’s neighbours. They may have thought Browne’s prophetic announcements were about an impending natural death but, after he killed himself, reinterpreted them—perhaps with some guilt—in light of his suicide. This is indicative of the tension within the statements themselves, which envisage both a natural and a suicidal death; the two are linked, as bodily decline motivated, and even presaged, Browne’s suicide. Nowhere is this clearer than in Browne’s statement to another neighbour, that ‘he would not hop into a Coffin [i.e. kill himself] but God knows how soon he may be laid in one’.^52^ Although he did kill himself, this indicates that Browne felt, as an ailing older man, overwhelmingly weighed down by, and perhaps resigned to, death’s ever-increasing imminence.

In his study of physical disability, David Turner notes that work played ‘a direct role in establishing men’s identity’. ^53^ In Browne’s case, it is significant that his declining occupational powers generated ideas about ‘lameness’ and mortality. It should be noted that 75 per cent of the 106 suicides examined here were carried out by men, and male anxieties about declining occupational abilities were extremely significant.^54^ Differences in late-life occupational stresses do not account for all of the gender disparity—it seems, for one thing, that men potentially chose more lethal methods of suicide—but they are an important part of the story.^55^ Worries about declining employability, expressed partly through ideas about ‘lameness’, were present in the case of Isaac Hendley, ‘an elderly Man between Sixty and Seventy years of age’ who cut his throat in Shoreditch in 1797. Hendley often ‘express[ed] his apprehension that he should come to want’ and ‘that he should be incapable of working’ as a shoemaker—he was ‘afraid he should be … passed to his parish’. Parish dependence was potentially shameful for a once self-reliant man, since financial and social independence were valued as key attributes of eighteenth-century masculinity.^56^ It should be noted that, as late as 1817, over 80 per cent of employed men worked in the primary and secondary sectors—from agriculture and mining, to shoemaking and building—with most of these jobs requiring strength and/or dexterity, qualities which generally declined in later life.^57^ Hendley’s anxiety arose because he ‘complained of several bodily infirmities’, particularly ‘in his Limbs [and] used to say he was afraid he should lose the use of his Limbs’.^58^ Notably, many of his concerns were about decline and his near-future state—he was ‘afraid’ of being passed onto his parish, and ‘afraid’ of losing the use of his limbs, with the language of ‘loss’ being present both here and in the words of Browne above. Hendley was also ‘an outpatient of St Bartholomew’s Hospital … on account of the veneral Complaint’ for, although ‘his Memory and recollection failed him very much’, he attributed certain problems to ‘having had the Veneral Disease’ in his past.^59^ As his friend and colleague explained, due to the accompanying pains, Hendley took ‘Opium to make him sleep’ at night; Hendley thus turned to self-medication before eventually killing himself. He had struggled immensely through the past 12 months of his life, ‘often wish[ing] himself dead’, and clearly saw little future in his deteriorating body.^60^

Older suicidal people often expressed such feelings about the irreversible direction of bodily decline, and its relation to dependency and degradation. William Richmond, an ‘old Carpenter’ who hanged himself in Westminster in 1790, was deeply affected by his dwindling bodily powers and what they represented. He had ‘become Ill and Lame’ and was accordingly ‘in want’, encumbered with a body that no longer ‘worked’, in multiple senses of the word.^61^ His son’s servant reflected that he ‘was not in the Least Surprized’ that Richmond had killed himself, because ‘having formerly lived well and Come to decay very much effected his Mind’.^62^ The idea of ‘decay’ is notable here, holding connotations of both bodily and economic deterioration; similar language is, as Steven King notes, found in old people’s pauper letters from a similar period, where elders drew upon the notion of ‘decay’ not only to engender compassion, but also to indicate that ‘they would not likely be a long-term burden on the parish’.^63^ This relationship between physical and economic health appears, too, in James Nicholas’ inquest. Though he had been melancholy for 17 years, Nicholas, an older labouring man who hanged himself in Suffolk in 1792, had become particularly fixated on his ‘very Bad Ancle’, which made him ‘at times burst into Tears and say he feared he never should be well again and should come to want’.^64^ Here, again, were fears about permanency, about never being ‘well again’. Importantly, when facing such worries about agedness and work, older people could be ‘rational’ and grounded in justifying their suicides. Cuthbert Cousins, who cut his throat in 1772, was ‘about Sixty Years of Age and infirm, & unable to work and maintain himself as he had done’.^65^ When his neighbour’s servant—who first found Cousins badly wounded but still ‘sensible’—asked him ‘how he came to do that Act’, Cousins simply ‘answered that being an old Man and out of work, he did it for want’, a forthright statement about the relationship between his age and occupational distress.^66^ For people like Cousins, and Hendley and Nicholas too, suicide was not a ‘medicalised’ act, driven by lunacy, but a rational response to the struggles of a burdensome and wretched old age, one that weighed particularly hard upon men who were socialised to find pride in being self-reliant breadwinners.^67^

The anxiety and debasement that late-life unemployment could bring were also clearly articulated by Thomas Emperor, an older ‘under Porter’ who was found drowned in 1799. Despite copious evidence of mental distress, the verdict was unusually given as ‘death by misfortune’, probably because Emperor worked for the Prince of Wales at Carleton House, and it was necessary to avoid a scandal.^68^ It is worth, however, paying attention to Emperor’s words, because they capture the deep, bodily unease which the threat of late-life unemployment could engender. As his colleagues testified, Emperor was very concerned about being dismissed; he feared

he should be turned out of his place and that then he should die in a Ditch like a Dog and be buried in a Ditch like a Dog … the deced soon after sayed there pointing to a Corner of the Room there is his highness and major Lee talking about me I am sure I shall be turned out of my place.^69^

The striking language and debased imagery of dying ‘in a Ditch like a Dog’—which, being repeated, was possibly Emperor’s phrase—indicates feelings of animal-like degradation and corporeal helplessness. It is worth noting that the decade within which the largest number of suicides occurred (over a quarter) was the 1790s, a time of considerable economic distress and employment vulnerability, which would have been particularly difficult for older people.^70^ In Emperor’s words, late-life dismissal and unemployment were closely linked to death, just as old-age illnesses and problems were, in the other inquests above, connected to deterioration and mortality.

As Hannah Newton notes, the weakness and decrepitude of old age were thought to present hindrances to recovery from illnesses and physical problems; Daniel Schäfer even argues that old age was pathologised as a unidirectional ‘state of illness’ in the eighteenth century.^71^ This seems, overall, to be how many of these elders conceptualised their agedness. Thomas Norman, a gentleman who killed himself in St James’s in 1771, was, according to his apothecary, ‘upon every illness … effected by a great Dejection of Spirits’, as if each one represented another aspect of his decline.^72^ Notably, Norman left this apothecary £1000 in his will, a sum which not only indicates the extent of the care he received before he died, but perhaps that he felt no resentment towards the man who ultimately failed to relieve his problems.^73^ There was, arguably, an understanding that some illnesses or age-related bodily issues could not be cured. Accordingly, there is a sense, in all of these cases, that the time that stretched out before these men was irreparably bleak, an idea which was expressed in Sir Roger North’s *Notes of Me*, written around the turn of the eighteenth century. As he mused, when men are ‘desperate in health … as in [the] case of wretched old age … so that death is certain, but the time uncertain, only the acute pains and comfortless condition in the interim infallible … I admire that any man in such condition should desire to live, or indeed should not court death’, or even ‘wilfully procure it’.^74^ It was as if these elders were living in an ‘interim’ between life and death, observing their bodies deteriorate before them.

## Embodied Memory-Loss

The relationship between bodily deterioration and emotional or mental suffering was not unidirectional, with a ‘mind’ responding to a declining body. Minds are always embodied, and old-age somatic decline affected memory and cognizance in distressing ways. Older suicidal people were particularly likely to be affected by memory-loss, which is mentioned in 19 per cent of the 106 cases examined here, compared with 4 per cent of cases in the general sample.^75^ Few historians have looked at memory-loss in this period. Daniel Schäfer has discussed ideas about it in early modern Europe, using published medical, theological and literary texts.^76^ G. E. Berrios has similarly discussed educated conceptualisations of memory-loss—using encyclopaedias and medical works—as has, to some extent, Patrick Fox, François Boller and Margaret Forbes, in cursory preambles to discussions about modern dementia.^77^ Few historians have, however, looked at old-age experiences of memory-loss, most likely because of the difficulty of locating source material. The inquests offer us ways into examining these experiences, allowing us to look at the angry and despairing feelings which a ‘declining mind’ could engender.

Take the case of John Braithwaite, an elderly gentleman who drowned himself near Egremont in 1803. Braithwaite was so frail that he asked one of his servants to kill him. As she deposed, he ‘complained much of uneasiness and being afflicted and sayd there must be an End’, and ‘asked her if she wd. put an End to him—He sayd he cd. not do it himself’.^78^ He suffered most, however, with memory-loss and confusion; he ‘complained of an uneasiness and confusion in his head and Giddiness and want of recollection’, and of feeling frustrated that ‘he could not recollect the most Common Words and could not express what he wanted to say’.^79^ John Jackson, his surgeon, said that when he visited Braithwaite, he often had a ‘Vacant stare as if he did not immediately recollect [him]’ and concluded that he had an ‘Affliction of the Brain’, but ‘from the Deceased’s time of Life he thought it not very probable that he wd. ever recover perfectly’. He thought it ‘wd. prove in all probability the Cause of Death tho’ not in the unhappy way it has now done’.^80^ These comments indicate that Braithwaite was in ‘decrepit old age’, for not only was he very frail, but his ‘time of Life’ is drawn upon to indicate the irreversibility of his mental decline.

In the inquest, we can thus see how those around Braithwaite observed his deterioration, identifying embodied memory-loss as crucially important to his demise. Note how Jackson located Braithwaite’s problems in a declining ‘brain’; as physician David Hartley’s 1801 edition of *Observations on Man* taught, in cases of old-age dotage:

the brain itself [is] in a languishing state … we see, in old persons, all the parts, even the bones themselves, waste, and grow less. Why may not this happen to the brain … to destroy the powers of association and memory?.^81^

Schäfer has argued that, after 1700, medical writers increasingly ‘somaticised’ memory-loss—that is, located its cause in somatic decline and linked it to old age—a trend that might explain Jackson’s discussion of cerebral deterioration. ^82^ It may also, more generally, explain the higher chance of later inquests to feature discussions of memory-loss, from both medics and laypeople. Just as Newton has shown that early modern doctors had some conception of ‘children’s physic’ and the specific needs and characteristics of children’s bodies, Jackson’s observations about Braithwaite’s brain and his ‘time of Life’ indicate that doctors were aware of some of the limitations of old bodies.^83^

In the depositions we also, however, get a sense of Braithwaite’s own emotional response to his memory-loss. He articulated his anguish to his servant, saying that ‘he thought it hard that the Almighty shd. afflict him in that manner’, and to his surgeon, ‘complain[ing] that his situation was dreadful and Death wd. be a Blessing to him’.^84^ It is interesting that, while his surgeon located Braithwaite’s decline in his ageing brain, Braithwaite turned to an older language of providence, seeing God as the source of his affliction. Eventually, it became difficult for Braithwaite to lead a normal life and participate in his community as before; he had been a respected and prosperous man, owning multiple properties and even a pew in Egremont Church.^85^ When he went fishing with his friend, he ‘quite lost his recollection’ and ‘did not know how he had got home nor what he had done with, nor where he had left, his Horse’, something that deeply unsettled him. His horse was not only necessary transportation, but also an important gentlemanly status-symbol. In response to these problems, Braithwaite sometimes lashed out; when he and his companions were playing cards, he ‘broke out into a most Violent Frenzy without any cause’ and ‘cursed (which he was not used to do) most Violently and behaved in so Frantic a Manner that [his friend] was obliged to Carry him home’.^86^ This uncharacteristic outburst shocked his friends—Braithwaite was not only increasingly unable to recognise his companions, but they were becoming less able to recognise him, as if his former identity was being eroded by somatic change. Braithwaite’s anger arguably related to the confusion—and therefore frustration—that he was experiencing through memory-loss; anger and/or aggression are well-observed among elders suffering with dementia in the present day.^87^ However, in getting so angry, there is a sense in which Braithwaite was losing his grip of the affective behaviours endorsed by his ‘emotional community’; the seeming randomness and inappropriateness of his angry outbursts made it difficult for others to understand him.^88^ These incidents point to the complexity and range of Braithwaite’s emotional responses as he was confronted, in various contexts, with his declining mind.

A wavering memory thus lessened elders’ independence and ability to function within their communities. Nevertheless, Jesse Cook, an ailing older man who lived in rural Suffolk, tried, like Braithwaite, to behave ‘normally’ as before. Repeatedly, Cook set out for one familiar destination, but ended up at another, as he ‘turned into the fields, lost himself’ and resurfaced late at night. When he went to fetch water, his housekeeper, becoming nervous that he might ‘tumble, or throw himself into the well’ tried to help him, but he ‘would not let her’, indicating that Cook felt aggrieved at his lessening independence.^89^ Despite being a ‘man who always spoke very much against any man’s taking away his own life’, Cook eventually hanged himself.^90^ Living in a society which condemned suicidal behaviour, people often had to justify their actions to themselves, but the combination of physical problems and worsening mental powers could present an ‘interim’ of continuous suffering. Such was the case with George Heming, an ailing goldsmith who poisoned himself in Westminster in 1783. Heming was affected by agonising physical problems—his ‘violent Scorbutic Disorder’ affected his stomach, and produced ‘Irruptions upon his Arm, which made the Deced. uneasy’—but he had also ‘lost his Memory and was in capable of doing Business’.^91^ With this combination of bodily and mental deterioration, Heming had rationalised his act; when his apothecary, George Ireland, said ‘how wrong it was to take any thing that might destroy him’, Heming ‘only said that he had Settled that with himself’.^92^ For Heming, his suicide was an understandable decision, the morality of which he had ‘settled with’ himself. Notably, Heming’s servant noted that Heming had ‘t[aken] Medicines from Mr Ireland the Apothecary but … did not grow better but rather worse’; perhaps Heming was not looking to be lectured by a medic who had only increased his suffering.^93^

As with Cook, confusion and memory-loss could disrupt older people’s sense of place, both geographically and temporally. James Barkley, who hanged himself in his cellar in 1776, became disorientated in the years before his death, often commenting to his friend that ‘he cod. not tell whether he was at Arkill's (meaning the Queen's Head [an alehouse]) or at home’.^94^ Robert Lovell, who hanged himself in London in 1772, could become similarly spatially confused. Lovell—who had been coachman to the Duchess of St Albans—was, when driving, twice ‘Seized in the Street and could not tell where he was, though quite sober’; as a colleague explained, this elicited a deep despondency, and ‘he was unable to drive the Dutchess … [so] she has lately hired another Coachman, the Deced from his lowness of Spirit and great Age being unable to drive’. ^95^ Lovell’s case highlights how senility could, just as much as bodily infirmity, impact upon employability and independence, and also how it was explicitly linked, in others’ minds, to ‘great Age’. In a different way, before her death, Martha Fuller—a weaver’s wife who hanged herself in 1792—seemed, in old age, to lose track of temporal placement, and to return to memories of an earlier period of mental distress. As her husband explained, she had, ‘about twenty years past … the Misfortune to loose a Child’, something ‘which affected her Brain so much’ that she had attempted ‘to destroy herself’ at the time.^96^ As John Haslam wrote in 1809, it was often the case that, in old age, ‘the transactions of the latter part of life are feebly recollected, whilst the scenes of youth … remain more strongly impressed’; perhaps, in her advancing years, Fuller was tormented by this sorrowful, long-ago remembrance.^97^ These disruptions show how declining minds could become dislocated from the worlds in which they existed. Pain and infirmity, and memory-loss and senility, could have a devastating impact upon older people’s emotional wellbeing, particularly, it seems, upon those in the latter stages of old age.

## Burdensome Age

In advancing years, physical and mental ill-health took a toll on people’s sense of personhood. There is an impression, in the sources, that a life encumbered with age could itself be regarded as a burden. This idea was explicitly suggested in David Hume’s controversial *Of Suicide* (1777), in which Hume declared that when ‘age, sickness, or misfortune may render life a burthen, and make it worse even than annihilation’, suicide is ‘consistent with … our duty to ourselves’.^98^ In the inquests discussed so far, it is clear that many older people felt burdened by their age, as something that weakened their bodies and minds, and impacted upon their ability to work, with suicide being a method of escape. Age could also, however, make older people feel like burdens to those around them. Richmond was notably concerned not to be an encumbrance to his son in his old age. When his landlord’s wife ‘advised him to apply to his Son or go to an Hospital, he said he hoped he should be better and should wish not to trouble his Son’, and when his landlord similarly ‘advised him to send to his Son, he said he would Send to him if he did not get better’, clearly desiring to avoid or delay bothering him.^99^ As Ottaway notes, families felt a ‘moral obligation’ to support and care for their elderly members, but this did not mean that elders always felt able to receive this care.^100^

Indeed, the physical and financial care that older people often needed could make them feel ashamed and anxious. The case of James Dowdle, who cut his throat in Bath in 1803, encapsulates this. Dowdle had been committed to gaol, but was still being cared for by his daughter, who ‘went up to her father’s room to take away his breakfast things and his dirty linen when to her great astonishment and surprise she saw him lying on the side of his bed’.^101^ When he was asked what he had done and why, Dowdle only ‘said he hoped he had not much soiled the bed for that he endeavoured to keep it as clean as he could by lying near the side of the bed with his head out [so] that the blood might discharge itself into the pot which was by the side of the bed’.^102^ This seemingly unusual response is illustrative of a deep concern about being an inconvenience; given that his daughter regularly removed his dirty linen, there is perhaps some significance in Dowdle’s fixation on the cleanliness of the bed, as if he wanted to prevent more filial labour being created by his suicide. As Lisa Wynn Smith contends, in this period, ‘leaky male bodies’ were often considered disturbing and problematic, and it is notable that Dowdle worried that his blood would ‘soil’ the ‘clean’ bed.^103^ There were strong connections between clean linen and social respectability, and dirtied linen or underclothes were associated with shame.^104^ In old age, bodies could of course become incontinent and ‘leaky’, and this different sort of leakiness could also elicit embarrassment. James Hilton, who hanged himself in Westminster in 1768, was cared for by his wife who, on the day before his suicide, found that he had ‘wetted his Shirt and the Bed in which they laid, (Which he frequently used to do)’, causing her to ‘ha[ve] some words in Anger with [him]’.^105^ Straight after, while his wife was fetching water, Hilton hanged himself. This scolding—which, on top of highlighting his body’s ‘feminine leakiness’, upset his role as male head of household—potentially impacted upon his decision.^106^

Of course, not all older people could be cared for by family members, and some consequently faced different feelings of rejection or despondency. Sarah Fenwick had been residing with her adult daughter since her husband died four months before, but, as her daughter testified, she ‘several times told her Mother that her Family was large and ha[d] perswaded her to go into the Workhouse’.^107^ Fenwick had solemnly replied that ‘she hoped she should be provided for soon’, perhaps suggesting that she wanted to be looked after by her family. Fenwick possibly felt uncared for; when her son had ‘treated her with a Pint of Beer’ the week before, she told the victualler that it was ‘very rare and unexpected’, indicating that she seldom felt thus indulged.^108^ Fenwick’s case highlights the issue of state-based care, and of wider societal responses to the ‘burden’ of ageing members. Siena has noted the poor’s ‘hatred’ of workhouses, which is attested in the sources examined here.^109^ They also, however, indicate that the workhouse could be especially stressful for ageing bodies. Workhouses housed many ageing people, with late eighteenth-century examples often having one-forth to one-third (or more) of their inmates aged 60 or over.^110^ Browne, the Essex man discussed above, articulated old-age anxieties about the workhouse very clearly; when his local victualler tried to ‘persuade him to go up to the workhouse again’, he said that ‘there was such a pack of little Devils [there], meaning the children, that he could not bear it, for he could not lay any thing down but that the Children would take it’.^111^ Browne thus experienced the workhouse as a place of intergenerational conflict, where he was hounded by ‘little Devils’ who used their youth to deprive him of food and other resources. As Ottaway notes, ‘workhouses were used disproportionally to house the young and the old’, a fact that could lead to such intergenerational antagonism.^112^ Browne’s experience was not dissimilar from Malthus’ 1796 characterisation of the workhouse, which he argued was ‘particularly hard upon old people', who ‘perhaps have been useful and respectable members of society’ but, ‘as soon as they are past their work’, are ‘obliged to quit the village where they have always lived, the cottage to which time has attached them, the circle of their friends, their children and their grand-children, and be forced to spend the evening of their days in *noise and unquietness among strangers*’.^113^

Most suicidal people thus viewed the workhouse as a wretched and even shameful place to spend one’s old age. This is suggested in the inquest of Thomas Coe, a 73-year-old man who killed himself in Saffron Walden in 1804. In the moments before he hanged himself, Coe—who was confined in the workhouse ‘Bridewell’—was interrupted by the workhouse tailor who, being threatened by Coe, ran off for assistance (which came too late). Before he ran off, the tailor had, however, observed Coe get ‘upon his Feet’ and say ‘he was ashamed of his Life and to live’, which were seemingly his last words.^114^ For Coe, life itself was a shameful burden, and living it a trial too great for him to bear.

Suicidal older people sometimes utilised, more explicitly, discourses around burdensomeness and the ‘bearing’ of loads. Before she killed herself in Maidstone in 1779, Phillis Jury rather cryptically expressed to her neighbour that ‘her Burthen was too great to bear’ and, on being asked why she had taken poison, replied that ‘she was tired of life’, ideas of downtroddeness and exhaustion connoted in both statements. Similarly, Ann Tiffin ‘Complained heavily of the Burthen she had upon her Mind’—a feeling that she ‘could never be happy in this world since the Death of her Husband’—as if her grief was a weight pulling down upon her.^115^ Of course, in these statements, it is difficult to distinguish the line between literality and metaphor, but it is reasonable to suggest that there is an element of embodiment here too. It seems that weightfulness could be expressed in ageing, suicidal bodies. Robert Lowthian, an elderly yeoman who hanged himself in Cumbria in 1718, had, on the night before he died, told his wife that ‘his heart was heavy & … that she was all his Comfort’, as if his sadness was a burden encapsulated in his chest.^116^ Expressions of weightfulness are not exclusive to older suicidal people, but it is worth considering how multiple discourses of burdensomeness were layered onto the body of a man like Lowthian, who was encumbered with declining physical abilities and a dwindling power within his community. Similar language can be found in older people’s pauper letters, with William King writing, in 1834, that he felt ‘Great weakness and Sinking Such as I am perswaded all People feel who are told 60 years’; ‘Long Years of Trubble have Bowed Me down as it wheare’.^117^ Like in the case of Browne, who claimed that ‘age and Infirmity had *reduced* him’, there was a sense that older people were shrunken, weighed down, dried out and diminished.^118^ Older people, characterised by the physician John Smith as having a tendency towards a ‘heaviness of spirits’, could be, or feel like, a ‘burden’ to others, but could also feel burdened and weighed down themselves.^119^

## Withdrawing Presence

It is within this context of corporeal and social problems that we turn to the issue of older suicidal people’s withdrawal from aspects of family or community life. Ottaway has argued that, though it was acceptable for (wealthy and self-sufficient) elders to ‘withdraw from their life’s work’, overall, older people remained active participants in their communities.^120^ Given this expectation, it could then be devastating when elders felt unable to partake in community life. This is captured in the experience of Lowthian who, having once been an important and respected figure in his village, felt reduced in his old age; when his neighbour asked him for some guidance about his farm affairs, Lowthian replied that ‘he was not so able to give advice’ anymore, suggesting that he was losing his place as an expert in his community network.^121^ He had also previously told his son that ‘if it was nt. for the sake of his family he would put an end (to himself)’, but sadly, in his old age, these social ties were ultimately unable to preserve him.^122^

Part of this withdrawal of presence arose from ‘regressions’ in behaviour, which made it difficult for others to understand older people. It was a common trope that the elderly entered a ‘second infancy’; as George Cheyne wrote, ‘old Men … *become, as it were*, Children a second time’.^123^ Margaret Hay, aged 78, was said to be ‘very childish and melancholy in her mind’, often ‘express[ing] herself very unreasonably’, while William Woodcock, aged ‘between Seventy and eighty Years old’, was ‘at times very Childish and as if he was deranged in his Sences’, often talking to himself’ but ‘never answ[ering] when spoke to’.^124^ Notably, references to ‘childish’ behaviour only appear in inquests involving those aged over 70, indicating that an infantile dotage was perceived to occur at a particularly late stage of life; as one pauper letter-writer notably contended in 1825, his wife ‘is become a child which we may expect she being in her 79 or 80th year’.^125^ It is extremely difficult to know how people like Hay or Woodcock experienced this sort of senility, as it was bound up with breakdowns in the communicative behaviours necessary for their thoughts to be translated into the archival material. Such is the intrinsic problem in discussing the experience of withdrawal, in which the silences and miscommunications often have to stand in for the normative interactions which are usually reported.

The withdrawal of communication is significant in itself, and some elders seem to have made a more conscious effort towards it. Before her death, Elizabeth Sharp, an older woman who killed herself in London in 1813, stopped engaging with her family, ‘refusing to see any visitors & if accosted on the stairs … would return no answer’.^126^ She also behaved in ways which confused them; shortly before her death, she ‘put on a child’s bonnet & went out & was away for about 3 or 4 hours & none of her family knew where she had been’.^127^ As Helen Yallop has argued, a cheerful sociability was thought to promote longevity and the health of the ageing body, so this withdrawal was deeply worrying for families, and often commented upon.^128^ To the distress of his family, John Bucknell, particularly when he was sober, would ‘lock himself up in a sulky humour for two or three Days together during which time he would speak to no one’ and ‘used at these times to refrain from either eating or drinking’.^129^ He thus retracted not only his communication, but his physical presence. It is possible that Sharp and Bucknell, in these withdrawals, were preparing for their suicides, their ultimate departures. It is worth noting that suicides were often described as occurring behind ‘locked doors’, with families and neighbours frequently having to knock them down in order to discover what had happened. Therefore, solitude was tied up with suicidality, and held ominous connotations.^130^ It was an indication of mental distress in itself, as in the case of the ailing Robert Rose, who was said to have appeared ‘very ill and uneasy in his mind by sitting and walking about alone musing’.^131^ Again, these behaviours seemed to alienate elders’ from their emotional communities which, as is clear from the inquests, valued certain levels of positive or at least neutral social interactivity.

Some elders had a rather different relationship with solitude, and were afraid of being alone. This fear was still, however, connected with a reduced or withdrawn presence, in the sense that it often stemmed from feelings of physical and social vulnerability. While some historians—such as Keith Snell and Fay Bound Alberti—have paid increased attention to the historical issue of loneliness, few have discussed this connection between fear and aloneness in the vulnerable.^132^ In these cases, it often seemed to arise at night, a time of particularly decreased sensory capacity and removal from the purview and daytime activities of others. William Buttram, an older man who drowned himself in Suffolk in 1804, asked for another lodger to sleep with him, because ‘he could not rest if he slept alone’; on the night before he died, he had also ‘asked him to light a piece of candle’ when they went to bed, perhaps because it made him feel safer.^133^ Interestingly, the elderly Braithwaite, discussed above, similarly requested that his servant leave ‘a small Candle in the Room which had been used to Burn by him for a few night last Past’ even though it was ‘not his Common practice’, suggesting that he was feeling particularly vulnerable or scared in the period before his suicide.^134^ At night, his door was also ‘left open by his desire, he ordered it not to be Shut’, and his two servants were asked ‘to sit up all Night to give the Deceased anything he was in need of’, indicating not only that he required physical assistance, but also that he might have felt safer knowing that he was never alone.^135^ Older people were often dependent upon the care of those around them, sometimes leading to fears of what would happen if they were solitary.

Some of this fear seemingly stemmed from older people’s concerns about what they might do to themselves. Indeed, while I have shown that many older people saw their suicides as a rational response to the problems of agedness, there were also some cases in which older suicidal people felt mentally unwell, and were afraid of their own capabilities. Elizabeth Hughes, who had been ‘Infirm in body’ for a few years, had told her nurse that she was troubled by ‘very bad thoughts and was afraid of being alone’, while Margarett Leverett, a 55-year-old woman who killed herself in Saffron Walden in 1783, was similarly ‘afraid to be left alone’.^136^ Notably, Leverett also occasionally ‘talked upon the Subject of Destroying herself’ and ‘sayed she was afraid her disorder was coming again & cried’, statements which suggest that she thought there was degree of inevitability in her self-inflicted death.^137^ Older people’s fears or beliefs in the inevitability of their death also tie into the notion of withdrawal, in the sense that agedness was connected with the departure from life itself. As popular ‘life-stage’ poems evidence, old age was thought of as a time of preparation for death; as *The Age and Life of Man* (c.1800) had it:

Then Man appears fifty five years,

And sick both Ev’n and Morn:

Loins, Legs and Thighs with sore Disease,

Make him to Sigh and say,

Ah! Christ on High have mind of me,

And Learn me for to die.’^138^

Indeed, in their agedness, older suicidal people certainly thought about the proximity of death, as in the case of Browne, discussed above, who said ‘he would not hop into a Coffin (i.e. kill himself) but God knows how soon he may be laid in one’.^139^ Joseph Fisher, an elderly man who killed himself in Cumbria in 1776, had similar concerns; before his suicide, he was ‘very much Depressed & Sunk’, and ‘talked frequently of his dying’.^140^ This sense of departure, or withdrawal into another world, is particularly evident too in the case of John Abbott, ‘an Old Man about sixty two years of Age’, who killed himself in London in 1799. Before Abbott’s death, he used to get up in the night and ‘cry out and mutter and would say “Oh here is the Devil has got hold of me; pray for me pray for me”’ and, when he was dying, shouted to his neighbours: ‘there is the Devil—let me go—I am going’, as if he could sense himself passing into a hellish afterlife.^141^ Abbott clearly felt fearful about death and, particularly, a suicidal departure—the devil was traditionally thought to instigate suicides.^142^ However, when he was dying, he also seemed to be readied for death, asking that people would ‘let me go—I am going’.^143^ Notably, Abbott was so severely infirm that ‘he never was able to get out of his Bed only while the Bed was [being made]’, a spatial constraint that not only ‘represented sickness’, but also cut him off from much of the social happenings of the household, potentially leaving him dislocated.^144^ Overall, the complexity of withdrawal is captured in the case of Abbott who, like Browne and Fisher too, was worried about the perceived imminence of death, but also ultimately the cause of it. The withdrawal of presence was a deeply multifaceted aspect of older suicidal people’s experiences, connected to issues of ‘regression’, status-loss, solitude, vulnerability and the suicidal act itself.

## Conclusion

It is surprising that scholars have not examined, in any depth, the reasons why older people were ‘unusually suicidal’ in this period.^145^ This article has shown that older people’s suicidal experiences are best understood through the lens of the ‘ageing body’, for it allows us to appreciate the complex interplay of issues of physical decline, mental deterioration, social withdrawal and the demotion of status. Indeed, those in the latter part of their lives struggled with a unique set of difficulties which are impossible to understand without a more nuanced understanding of how age shaped embodiment and societal and familial standing. Physical decline left older people feeling ‘reduced’ and vulnerable, subject to ever-worsening health and, ultimately, nearer to death itself. Mental decline—and particularly memory-loss—was an especially distressing experience, which provoked feelings of anger, confusion, diminishment and despair. Elders could see themselves, and be perceived as, a burden to their employers and families, while old age could itself manifest as a burden to the bodies encumbered with it. Lastly, older people could be pushed to the peripheries of the familial communities they may once have led, withdrawing from social interaction and, ultimately, the ties that bound them in life. Overall, these difficulties, frequently layered upon each other, produced a context in which older people were around two times more likely to kill themselves than the general population.

This article has aimed to show that coroner’s inquests provide a ‘way into’ examining the suicidal experiences which have been so long ignored by the traditional historiography. In the absence of other sources, the depositions of friends, families, neighbours and medical practitioners provide rich evidence of the behaviour, words and feelings of the ‘voiceless’ dead. Though not an unproblematic source, they clearly document complex and visceral feelings of distress, anger, fear, debasement, pain and despair, providing rich scope for emotions-history research which puts emotional actors at the centre of its narratives.^146^ Overall, this article resists the claim that the importance of the history of suicide lies ‘in what it reveals about the nature of cultural and social change’.^147^ Suicide should not just be used as an unusual or ‘extreme act’ with which to measure changes in society. The experiences of the suicidal are historically important in their own right, not just for the wider issues—surrounding, say, the scope of welfare provision—which they inevitably highlight. Rather, it is important to understand what it felt like, and what it meant, to be suicidal in the long eighteenth century. When we recentre the perspectives of the suicidal, we realise that, rather than being ‘medicalised’ or experienced as a product of lunacy, suicide could be felt as a rational response to the struggles of ageing, an end-point to bodily and mental suffering that medicine could not remedy.

## Funding

This work was supported by the Arts and Humanities Research Council [grant number AH/L503897/1]; The Wolfson Foundation [no grant number, as it is a charity] and the Social History Society Conference Bursary.

## Footnotes

1 Edward Hearne, City of London Inquisitions, London Metropolitan Archives, 27 August 1795, LL ref. LMCLIC650080410. ‘LL ref.’ stands for London Lives reference, from Tim Hitchcock, Robert Shoemaker, Sharon Howard and Jamie McLaughlin, et al., *London Lives, 1690-1800* (www.londonlives.org, version 1.1, 24 April 2012) [last accessed 05/04/2021].

2
*Ibid*.

3
*Ibid.*, LL ref. LMCLIC650080411.

4 Victor Bailey, *This Rash Act: Suicide Across the Life Cycle in the Victorian City* (Redwood City: Stanford University Press, 1998).

5 Michael Macdonald and Terence Murphy, *Sleepless Souls: Suicide in Early Modern England* (Oxford: Oxford University Press, 1990), 251; Robert Houston, *Punishing the Dead?: Suicide, Lordship, and Community in Britain, 1500-1830* (Oxford: Oxford University Press, 2010), 355. Terence Murphy has written a brief piece on child and adolescent suicide; see ‘“Woeful Childe of Parents Rage”: Suicide of Children and Adolescents in Early Modern England, 1507-1710’, *The Sixteenth Century Journal*, 1986, 17 , 259–70.

6 Macdonald and Murphy, *Sleepless Souls*, 256.

7 323 suicides in total, with 19 ages not given. 40.2 per cent (122) were aged 50 or over, while 28.0 per cent (85) were aged 60 or over. This data is referenced by Macdonald and Murphy, and used to make the point that suicide was common among the elderly, but has been independently verified. As Macdonald and Murphy stopped at 1800, this article has also included the 73 inquests of suicides which occurred from 1801 to 1815. See Coroners’ Inquests, Norfolk Record Office, NCR 6a.

8 42.9 per cent (6 out of 14). See Coroners’ Inquests, Essex Record Office, D/B/2/OFF/3.

9 E. A. Wrigley and Roger Schofield, *The Population History of England 1541-1871* (Cambridge: Cambridge University Press, 1989), 216-8; 217-8.

10 Lynn Botelho, ‘Old Age and Menopause in Rural Women of Early Modern Suffolk’, in Lynn Botelho and Pat Thane, eds, *Women and Ageing in Britain since 1500* (Cambridge: Cambridge University Press, 2004), 1–64, 44. See also Pat Thane, *Old Age in English History* (Oxford: Oxford University Press, 2000), 21–7.

11 Susannah Ottaway, *The Decline of Life: Old Age in Eighteenth-Century England* (Cambridge: Cambridge University Press, 2004), 16–64; Pat Thane, ‘Social Histories of Old Age and Ageing’, *Journal of Social History*, 2003, 37, 93, 97–103; Devoney Looser, *Women Writers and Old Age in Great Britain, 1750–1850* (Baltimore: John Hopkins University Press, 2008), 9–10.

12 Bernard Lynch, *A guide to health through the various stages of life* (London: Printed for the author, 1744), 3.

13 Thomas Paine, *Rights of man. Part the Second. Combining Principle and Practice* (Dublin: Byrne, 1792), 127.

14 Mary Fenton, City of London Inquisitions, London Metropolitan Archives, 29 December 1791, LL ref. LMCLIC650040599.

15 Mary Pritchard, City of London Inquisitions, London Metropolitan Archives, 11 April 1798, LL ref. LMCLIC650110197. For more examples, see Mary Allenby, City of London Inquisitions, London Metropolitan Archives, 4 September 1799, LL ref. LMCLIC650120692 and John Swindell, City of London Inquisitions, London Metropolitan Archives, 5 August 1790, LL ref. LMCLIC650030555.

16 For more on this, see Sarah Toulalan, ‘Elderly years cause a Total dispaire of Conception’: Old Age, Sex and Infertility in Early Modern England’, *Social History of Medicine*, 2016, 29, 333–59, 341. Toulalan also notes that ‘chronological old age was consistently calculated as beginning at 50’.

17 I define ‘suicidal’ as relating to a desire to end one’s life. For an alternate view, see Åsa Jansson, ‘From Statistics to Diagnostics: Medical Certificates, Melancholia, and “Suicidal Propensities” in Victorian Psychiatry’, *Journal of Social History*, 2013, 46, 716–31.

18 See Róisín Healy, ‘Suicide in Early Modern and Modern Europe’, *The Historical Journal*, 2006, 49, 903–19, 903.

19 Macdonald and Murphy, *Sleepless Souls*, 2. See also Michael Macdonald, ‘The Medicalization of Suicide in England: Laymen, Physicians, and Cultural Change, 1500-1870’, *The Milbank Quarterly*, 1989, 67, 69–91 and Michael Macdonald, ‘The Secularization of Suicide in England 1660-1800: Reply’, *Past and Present*, 1998, 119, 165–70.

20 Houston, *Punishing the Dead?*, 2. See also R. A. Houston, ‘Fact, Truth, and the Limits of Sympathy: Newspaper Reporting of Suicide in the North of England, circa 1750-1830’, *Studies in the Literary Imagination*, 2011, 44, 93–108. For more examples of cultural examinations of suicide, see Jeffrey Watt, *From Sin to Insanity: Suicide in Early Modern Europe* (Ithaca: Cornell University Press, 2004); Donna T. Andrew, *Aristocratic Vice: The Attack on Duelling, Suicide, Adultery, and Gambling in Eighteenth-Century England* (New Haven, CT: Yale University Press, 2013); Donna T. Andrew, ‘The Secularization of Suicide in England 1660-1800’, *Past and Present*, 1998, 119, 158–65 and Terri L. Snyder, ‘What Historians Talk About When They Talk About Suicide: The View from Early Modern British North America’, *History Compass*, 2007, 5, 658–74.

21 Macdonald and Murphy, *Sleepless Souls*, 133.

22
*Ibid*. For an earlier discussion of this thesis, see Michael Macdonald, ‘The Medicalization of Suicide in England: Laymen, Physicians, and Cultural Change, 1500-1870’, *The Milbank Quarterly*, 1989, 67, 69–91.

23 Susannah Ottaway, *The Decline of Life: Old Age in Eighteenth-Century England* (Cambridge: Cambridge University Press, 2004), 16–64; Pat Thane, ‘Social Histories of Old Age and Ageing’, *Journal of Social History*, 2003, 37, 93–111, 93; 97–103; Botelho and Thane, eds, *Women and Ageing*; Mariana Labarca, ‘The Emotional Disturbances of Old Age On the Articulation of Old-Age Mental Incapacity in Eighteenth-Century Tuscany’, *Historical Reflections*, 2015, 41, 19–36, 20.

24 Most were found in the populous London boroughs, but other locations are well-represented. The numbers found in each were: Maidstone (4), Whitehaven (11), Chelmsford (6), Ipswich (9), Norwich (5), Bath (4), Cambridge (2), Canterbury (1), Middlesex (2), Westminster (44) and the City of London (18). Although there were many more age-relevant inquests in Norwich, the vast majority did not contain depositions, so cannot be included for our purposes here.

25 For 25.4 per cent (27 out of 106) specific ages are given; for most others, identifying words (such as ‘elderly’ or ‘old’) are present. In several others, other details (such as marriages lasting over 25 years, or friendships over 40 years) are indicative.

26 Often within one or two days.

27 Houston, *Punishing the Dead?*, 17.

28 Fisher, ‘The Politics of Sudden Death’, 25, 28.

29 Henry Care, *English* *Liberties: or, the* *Free-born* *Subject's* *Inheritance* (London: B. Harris, c. 1703), 177.

30
*Ibid*., 179; Thomas Pearce, *The* *complete* *Justice of the* *Peace* (London: Henry Lintot, 1756), 101.

31 Carol Loar, ‘Medical Knowledge and the Early Modern English Coroner’s Inquest’, *Social History of Medicine*, 2010, 23, 488.

32 Mark Jackson, ‘Suspicious Infant Deaths: The Statute of 1624 and Medical Evidence at Coroners’ Inquests’ in Michael Clark and Catherine Crawford, eds, *Legal Medicine in History* (Cambridge: Cambridge University Press, 1994), 64–86, 65.

33 Hitchcock, Howard and Shoemaker, ‘Coroners’ Inquests into Suspicious Deaths (IC)’, *London Lives, 1690-1800* (www.londonlives.org, version, 1.1 17 June 2012). Available at https://www.londonlives.org/static/IC.jsp#to [Accessed 10/04/20].

34 See Macdonald and Murphy, *Sleepless Souls*, 122–25.

35 56 (60.8 per cent) lower class (such as labourers and paupers); 26 (28.2 per cent) middling class (such as artisans, apothecaries and yeomen); 10 (10.9 per cent) upper-class (such as landed gentry and noblemen). This is somewhat reflective of the social structure of eighteenth-century UK, which for 1798 Robert C. Allen puts at around 66.0 per cent lower-class, 32.5 per cent middle-class and bourgeoise, and 1.3 per cent upper-class. Here the upper classes are greatly overrepresented, partly because their deaths were more financially and socially significant to those with power, so there was a higher chance of inquests into their deaths. See Robert C. Allen, ‘Class Structure and Inequality during the Industrial Revolution: Lessons from England’s Social Tables, 1688–1867’, *Economic History Review*, 2019, 72, 88–125, 105.

36 Bailey, *This Rash Act*, 5. See Vicky Holmes, *In Bed with the Victorians: The Life-Cycle of Working-Class Marriage* (London: Palgrave Macmillan, 2017) and Elizabeth Hurren and Steven King, ‘Courtship at the Coroner's Court’, *Social History*, 2015, 40, 185–207.

37 Olive Anderson, *Suicide in Victorian and Edwardian England* (Oxford: Clarendon Press, 1987), 107–90.

38 Bailey, *This Rash Act*, 6. For an extended examination of the value of using inquests in this way, see 39–84.

39 John Weaver, *A Sadly Troubled History: The Meanings of Suicide in the Modern Age* (Montreal: McGill-Queen’s University Press, 2009), 162–344. Inquests have also been used to look at other life experiences, such as ‘the experience of marriage and marital relations’; see Holmes, *In Bed with the Victorians*, 2.

40 Vera Lind, ‘The Suicidal Mind and Body: Examples from Northern Germany’ in Jeffrey Watt, *From Sin to Insanity: Suicide in Early Modern Europe* (Ithaca: Cornell University Press, 2004), 64–80, 67–68.

41 E. T. Isometsä, ‘Psychological Autopsy Studies – A Review’, *European Psychiatry*, 2001, 16, 379–85, 379.

42 Rob Boddice, *A History of Feelings* (London: Reaktion Books, 2019), 189.

43 Barbara H. Rosenwein, *Emotional Communities in the Early Middle Ages* (Ithaca: Cornell University Press, 2006), 25–26.

44 They are listed sequentially, in the order that they appear (excepting repetitions).

45 Thomas Knagg, *A* *Sermon* *Against* *Self-murder* (London: J. Read, 1708), 5.

46 ‘Rationalist’, *Duelling and suicide repugnant to revelation* (London: 1774), 24.

47 Kevin Siena, ‘Suicide as an Illness Strategy in the Long Eighteenth Century’ in John Weaver and David Wright, eds, *Histories of Suicide: International Perspectives on Self-Destruction in the Modern World* (Toronto: University of Toronto Press, 2009), 53–72.

48 Less than 20 per cent (139) of the total 716 subjects were said to be affected by physical problems, compared with 34 per cent (36) of the 50-and-over sample.

49 Note that multiple themes could occur in a single inquest.

50 Underlined by the coroner. Thomas Browne, Chelmsford Inquisitions, Essex Record Office, 12 March 1802, D/B 2/OFF3/50.

51
*Ibid*.

52
*Ibid*.

53 David Turner, *Disability in Eighteenth-Century England: Imagining Physical Impairment* (New York: Routledge, 2012), 128. See also Alannah Tompkins, ‘Labouring on a Bed of Sickness’: The Material and Rhetorical Deployment of Ill-health in Male Pauper Letters’ in Andreas Gestrich, Elizabeth Hurren and Steven King, *Poverty and Sickness in Modern Europe: Narratives of the Sick Poor, 1780-1938* (London: Continuum, 2012), 51–68, 54.

54 79 out of 106 (74.5 per cent). This is typical of overall gender ratios for suicide in this period.

55 Today, it is well documented that women attempt suicide more than men, but men more often die. See Diane Currier and Maria A. Oquendo, ‘Epidemiology of Suicide and Attempted Suicide’ in Ming T. Tsuang, Mauricio Tohen and Peter Jones, ed., *Textbook of Psychiatric Epidemiology* (Chichester: John Wiley & Sons, 2011), 517–34, 520. We do not have good statistics for attempted suicides in this period, but the gendered differences in suicide method points to men choosing more lethal options. For example, within the older-age sample, nearly three times as many women (7.4 per cent to 2.5 per cent) chose poison, and considerably more (22.2 per cent to 13.9 per cent) chose drowning, both methods which present slightly more scope for recovery. No women chose shooting, compared to 7.6 per cent of men, and considerably more men (24.1 per cent to 14.8 per cent) chose throat-cutting, particularly dangerous methods.

56 See Matthew McCormack, *The Independent Man: Citizenship and Gender Politics in Georgian England* (Manchester: Manchester University Press, 2005).

57 E. A. Wrigley, *The Path to Sustained Growth* (Cambridge: Cambridge University Press, 2016), 69.

58 Isaac Hendley, City of London Inquisitions, London Metropolitan Archives, 11 May 1797, LL ref. LMCLIC650100204.

59
*Ibid*.

60
*Ibid*.

61 William Richmond, Westminster Inquisitions, Westminster Abbey Muniment Room, 10 August 1790, LL ref. WACWIC652300490-1.

62
*Ibid*, 91.

63 Steven King, *Writing the Lives of the English Poor, 1750s-1830s* (Montreal & Kingston: McGill-Queen’s University Press, 2019), 291.

64 James Nicholas, Ipswich Inquisitions, Suffolk Record Office (Ipswich), 10 March 1792, HB/10/9/7/3.

65 Cuthbert Cousins, Westminster Inquisitions, Westminster Abbey Muniment Room, 4 April 1774, LL ref. WACWIC652120185.

66
*Ibid*, 86.

67 For more on this point and its relation to the body, see Joanne Begiato, *Manliness in Britain, 1760–1900: Bodies, Emotion, and Material Culture* (Manchester: Manchester University Press, 2020), 168–202.

68 Thomas Emperor, City of London Inquisitions, London Metropolitan Archives, 27 June 1799, LL ref. LMCLIC650120531. Due to the verdict, Emperor has not been counted in the 106.

69
*Ibid*., 33.

70 Samantha Williams, *Poverty, Gender and Life-Cycle Under the English Poor Law, 1760-1834* (Woodbridge: The Boydell Press, 2011), 36.

71 Hannah Newton, *Misery to Mirth: Recovery from Illness in Early Modern England* (Oxford: Oxford University Press, 2018), 46, 63; Daniel Schäfer, ‘“That Senescence Itself is an Illness”: A Transitional Medical Concept of Age and Ageing in the Eighteenth Century’, *Medical History*, 2002, 46, 525–48, 548. This idea is also supported by Amie Bolissian-Mcrae, ‘“A conservative cure in respect of Age”: The Contingent Nature of Recovery for Early Modern Ageing Patients’, paper presented at Reconsidering Illness and Recovery in the Early Modern World, University of Durham (Online), 18–19 August 2020.

72 Thomas Norman, Westminster Inquisitions, Westminster Abbey Muniment Room, 24 May 1771, LL ref. WACWIC652110247.

73 Will of Thomas Norman, Gentleman of Saint James Westminster, Proved 8 June 1771, NA, PROB 11/968/151.

74 Sir Roger North, Augustus Jesopp, ed, *The Lives of the Right Hon. Francis North, vol 3* (London: G. Bell, 1890), 151.

75 20 (18.9 per cent) out of 106; 28 (3.9 per cent) out of 716.

76 Daniel Schäfer, ‘Gulliver Meets Descartes: Early Modern Concepts of Age-Related Memory Loss’, *Journal of the History of the Neurosciences*, 2003, 12; Daniel Schäfer, ‘No Old Man Ever Forgot Where He Buried His Treasure: Concepts of Cognitive Impairment in Old Age Circa 1700’, *Journal of the American Geriatrics Society*, 2005, 53, 2023–27.

77 G. E. Berrios, ‘Dementia During the Seventeenth and Eighteenth Centuries: A Conceptual History’, *Psychological Medicine*, 1989, 17, 829–37; Patrick Fox, ‘From Senility to Alzheimer's Disease: The Rise of the Alzheimer's Disease Movement’, *The Milbank Quarterly*, 1989, 67, 58–102,60–65; François Boller and Margaret Forbes, ‘History of Dementia and Dementia in History: An Overview’, *Journal of the Neurological Sciences*, 1989, 158, 125–33.

78 John Braithwaite, Whitehaven Inquisitions, Whitehaven Archives, 5 December 1803, D/LEC/CRI/112/13.

79
*Ibid*.

80
*Ibid*.

81 David Hartley, *Observations on Man, vol. 1* (Warrington: J. Johnson, 1801) (Original 1749), 392–93.

82 Schäfer, ‘No Old Man’, 2026.

83 Hannah Newton, ‘Children’s Physic: Medical Perceptions and Treatment of Sick Children in Early Modern England, c. 1580–1720’, *Social History of Medicine*, 2010, 23, 456–74.

84
*Ibid*.

85 Will of John Braithwaite, Gentleman of Egremont, Proved 7 December 1803, Whitehaven Archives, DBH/2/24.

86 Curved-bracketed phrase in original. John Braithwaite, Whitehaven Inquisitions, Whitehaven Archives, 5 December 1803, D/LEC/CRI/112/13.

87 See Marc Hamel et al., ‘Predictors and Consequences of Aggressive Behavior by Community-Based Dementia Patients’, *The Gerontologist*, 1990, 30, 206–11, 206.

88 For more on this notion, see Barbara Rosenwein, *Generations of Feeling: A History of Emotions, 600-1700* (Cambridge: Cambridge University Press, 2016), 3.

89 Jesse Cook, Ipswich Inquisitions, Suffolk Record Office (Ipswich), 2 October 1815, HB/10/9/30a/12.

90
*Ibid*.

91 George Heming, Westminster Inquisitions, Westminster Abbey Muniment Room, 13 October 1783, LL ref. WACWIC652230532; 1.

92
*Ibid*.

93
*Ibid*.

94 James Barkley, Westminster Inquisitions, Westminster Abbey Muniment Room, 10 December 1776, LL ref. WACWIC652160520.

95 Robert Lovell, Westminster Inquisitions, Westminster Abbey Muniment Room, 16 May 1772, LL ref. WACWIC652120295-6.

96 Martha Fuller, Westminster Inquisitions, Westminster Abbey Muniment Room, 12 November 1792, LL ref. WACWIC652320753.

97 John Haslam, *Observations on Madness and Melancholy* (London: J. Callows, 1809), 61–62.

98 David Hume, ‘Of Suicide’ in *Essays on Suicide and the Immortality of the Soul* (London: James Decker, 1799) (Original 1777), 13.

99
*Ibid*.

100 Ottaway, *The Decline of Life*, 7.

101 James Dowdle, Bath Inquisitions, Bath Record Office, 12 September 1803, BC/4/1/2/91-93.

102
*Ibid*.

103 Lisa Wynne Smith, ‘The Body Embarrassed? Rethinking the Leaky Male Body in Eighteenth-Century England and France’, *Gender & History*, 2010, 23, 26–46, 27.

104 Alice Dolan, ‘Touching Linen: Textiles, Emotion and Bodily Intimacy in England C. 1708-1818’, *Cultural and Social History*, 2019, 16, 145–64, 145, 150.

105 James Hilton, Westminster Inquisitions, Westminster Abbey Muniment Room, 4 April 1768, LL ref. WACWIC652080122.

106 Smith, ‘The Body Embarassed?’, 30.

107 Sarah Fenwick, 31 July 1777, Westminster Inquisitions, Westminster Abbey Muniment Room, LL ref. WACWIC652170357.

108
*Ibid*, 56.

109 Kevin Siena, ‘Hospitals for the Excluded or Convalescent Homes?: Workhouses, Medicalization and the Poor Law in Long Eighteenth-Century London and Pre-Confederation Toronto’, *Canadian Bulletin of Medical History*, 2010, 27, 5–25, 7. This is not to say, however, that workhouses were universally reviled by their inhabitants, or that these inhabitants were downtrodden and powerless; for a more nuanced picture, see David Green, ‘Pauper Protests: Power and Resistance in Early Nineteenth-Century London Workhouses’, Social History, 2006, 31, 137–59; Jeremy Boulton and John Black, ‘Paupers and their Experience of a Georgian Workhouse: St Martin-in-the-Fields, 1725-1824’ in Jane Hamlett, Lesley Hoskins and Rebecca Preston, eds, *Residential Institutions in Britain, 1725-1970* (London: Pickering & Chatto, 2013) and Susannah Ottaway, ‘“A Very Bad Presidente in the House”: Workhouse Masters, Care, and Discipline in the Eighteenth-Century Workhouse’, *Journal of Social History* (April 2020).

110 Susannah Ottaway, ‘The Elderly in the Eighteenth-Century Workhouse’ in Jonathan Reinarz and Leonard Schwarz, eds, *Medicine and the Workhouse* (Woodbridge: Boydell & Brewer, 2013), 40–57, 42–43.

111 Thomas Browne, Chelmsford Inquisitions, Essex Record Office, 12 March 1802, D/B 2/OFF3/50.

112 Ottaway, ‘The Elderly in the Eighteenth-Century Workhouse’, 42.

113 Italics my own. Quoted in Richard M. Smith, ‘Ageing and wellbeing in early modern England: pension trends and gender preferences under the English Old Poor Law, 1650-1800’ in Paul Johnson and Pat Thane, eds, *Old Age from Antiquity to Post-Modernity* (New York: Routledge, 2003), 64–95, 90–91.

114 Thomas Coe, Chelmsford Inquisitions, Essex Record Office, 3 April 1804, D/B 2/OFF3/54.

115 Phillis Jury, Maidstone Inquisitions, Kent History & Library Centre, 10 May 1779, Md/JCi/80; Ann Tiffin, Whitehaven Inquisitions, Whitehaven Archives, 30 June 1763, D/LEC/CRI/72/4.

116 Robert Lowthian, Whitehaven Inquisitions, Whitehaven Archives, 20 February 1718, D/Lee/CR I, 27/4.

117 Quoted in King, *Writing the Lives*, 287.

118 Italics my own. Thomas Browne, Chelmsford Inquisitions, Essex Record Office, 12 March 1802, D/B 2/OFF3/50.

119 John Smith, *The portrait of old age* (London: E. Withers, 1752), 136–37.

120 Ottaway, *The Decline of Life*, 89, 98.

121 Robert Lowthian, Whitehaven Inquisitions, Whitehaven Archives, 20 February 1718, D/Lee/CR I, 27/4.

122
*Ibid*.

123 Italics in original. George Cheyne, *An essay on regimen* (London: George Strahan, 1742), v.

124 Margaret Hay, Westminster Inquisitions, Westminster Abbey Muniment Room, 12 October 1804, MS: no. 55; William Woodcock, City of London Inquisitions, London Metropolitan Archives, 28 October 1788, LL ref. LMCLIC650010230-1.

125 Quoted in King, *Writing the Lives*, 289.

126 Elizabeth Sharp, Westminster Inquisitions, Westminster Abbey Muniment Room, 29 June 1813, MS: no. 23.

127
*Ibid*.

128 Helen Yallop, *Age and Identity in Eighteenth-Century England* (London: Routledge, 2013), 89–95.

129 John Bucknell, City of London Inquisitions, London Metropolitan Archives, 29 July 1799, LL ref. LMCLIC650120605.

130 Angus Gowland, ‘The Problem of Early Modern Melancholy’, *Past & Present*, 2006, 191, 77–120, 94.

131 Robert Rose, Ipswich Inquisitions, Suffolk Record Office (Ipswich), 7 May 1799, HB/10/9/15/14.

132 See Keith Snell, ‘Agendas for the Historical Study of Loneliness and Lone Living’, *The Open Psychology Journal*, 2015, 8, 61–70 and Keith Snell, ‘The Rise of Living Alone and Loneliness in History’, *Social History*, 2017, 42, 2–28; Fay Bound Alberti, *A Biography of Loneliness: The History of an Emotion* (Oxford: Oxford University Press, 2019).

133 William Buttram, Ipswich Inquisitions, Suffolk Record Office (Ipswich), 30 August 1804, HB/10/9/20/18.

134 John Braithwaite, Whitehaven Inquisitions, Whitehaven Archives, 5 December 1803, D/LEC/CRI/112/13.

135
*Ibid*.

136 Elizabeth Hughes, Westminster Inquisitions, Westminster Abbey Muniment Room, 27 April 1772, LL ref. WACWIC652120246; Margaret Leverett, Chelmsford Inquisitions, Essex Record Office, 25 August 1783, D/B 2/OFF/3/29.

137
*Ibid*.

138 Anon, *The age and life of man, or a short description of his nature, rise and fall* (London: c. 1800). See also Yallop, *Age and Identity*, 111.

139 Thomas Browne, Chelmsford Inquisitions, Essex Record Office, 12 March 1802, D/B 2/OFF3/50.

140 Joseph Fisher, Whitehaven Inquisitions, Whitehaven Archives, 14 January 1776, D/LEC/CRI/85/1.

141 John Abbott, City of London Inquisitions, London Metropolitan Archives, 14 February 1792, LL ref. LMCLIC650050375.

142
*Ibid*.

143
*Ibid*.

144 See Hannah Newton, ‘Out of Bed, But Not Yet Abroad’: Spatial Experiences of Recovery from Illness in Early Modern England’, *Early Modern Literary Studies*, 2020, 19, 1–19, 19.

145 Macdonald and Murphy, *Sleepless Souls*, 256.

146 Other court documents have been well used in this research, such as in William Reddy, *The Navigation of Feeling: A Framework for the History of Emotions* (Cambridge: Cambridge University Press, 2001), 257–314.

147
*Ibid.*, 338.

## Notes

Ella Sbaraini is a second-year PhD student at the University of Cambridge. She received her BA in History (2018) and her MPhil in Early Modern History (2019) from the University of Cambridge. Her PhD is entitled ‘The Suicidal in England and Wales, 1700-1850’, and seeks to be a ‘suicide history from below’ which focuses on the experiences of suicidal people in the past. This paper began life as her MPhil dissertation.

